# Printed Silk Microelectrode Arrays for Electrophysiological Recording and Controlled Drug Delivery

**DOI:** 10.1002/adhm.202202869

**Published:** 2023-03-03

**Authors:** Nouran Adly, Tetsuhiko F. Teshima, Hossein Hassani, George Al Boustani, Lennart J.K. Weiß, Gordon Cheng, Joe Alexander, Bernhard Wolfrum

**Affiliations:** ^1^ Neuroelectronics – Munich Institute of Biomedical Engineering Department of Electrical Engineering TUM School of Computation Information and Technology Technical University of Munich Hans‐Piloty‐Strasse 1 85748 Garching Germany; ^2^ Medical & Health Informatics Laboratories NTT Research Incorporated 940 Stewart Dr Sunnyvale CA 94085 USA; ^3^ Neaspec—Attocube Systems AG Eglfinger Weg 2 85540 Haar Germany; ^4^ Chair for Cognitive Systems Department of Electrical Engineering TUM School of Computation Information and Technology Technical University of Munich Arcisstrasse 21 80333 Munich Germany

**Keywords:** additive manufacturing, cell recording, electrophysiology, inkjet, microelectrode arrays, microelectrodes, soft materials

## Abstract

The use of soft and flexible bioelectronic interfaces can enhance the quality for recording cells’ electrical activity by ensuring a continuous and intimate contact with the smooth, curving surfaces found in the physiological environment. This work develops soft microelectrode arrays (MEAs) made of silk fibroin (SF) films for recording interfaces that can also serve as a drug delivery system. Inkjet printing is used as a tool to deposit the substrate, conductive electrode, and insulator, as well as a drug‐delivery nanocomposite film. This approach is highly versatile, as shown in the fabrication of carbon microelectrodes, sandwiched between a silk substrate and a silk insulator. The technique permits the development of thin‐film devices that can be employed for in vitro extracellular recordings of HL‐1 cell action potentials. The tuning of SF by applying an electrical stimulus to produce a permeable layer that can be used in on‐demand drug delivery systems is also demonstrated. The multifunctional MEA developed here can pave the way for in vitro drug screening by applying time‐resolved and localized chemical stimuli.

## Graphical abstract

Printed soft microelectrode arrays (MEAs) made of silk fibroin (SF) films for recording action potential from Hl‐1 cell culture are presented. The tuning of SF by applying an electrical stimulus to produce a permeable layer that can be used in on‐demand drug delivery systems is also demonstrated.

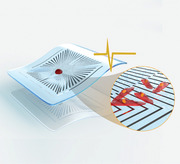

## 1 Introduction

Electrical tissue interfaces have a substantial influence in the field of bioelectronics and lie at the nexus of electrical engineering, biology, and material science.^[^ ^1^, ^2^, ^3^, ^4^, ^5^, ^6^ ^]^ Bioelectronic medicine is benefiting from the development of diagnostic and therapeutic techniques in treating diseases, including those of the heart, brain, and peripheral nervous system.^[^ ^7^, ^8^ ^]^ However, many of these procedures rely on stable in vivo signal modulation or monitoring using implantable electrodes. This has driven significant endeavors to enhance the interface between electrodes and biological tissue, with a focus on various aspects, including materials, geometry, size, and flexibility.^[^ ^4^, ^9^, ^10^, ^11^ ^]^ The design of bioelectronic devices faces a trade‐off between invasiveness and selectivity. Typically one distinguishes between penetrating probes, and probes which are deployed on the surface and do not penetrate the tissue.^[^ ^12^ ^]^ Consequently, the goal in this field has been the development of flexible electrode interfaces that can gather high‐quality electrical readouts while being embedded seamlessly in the biological environment without damaging the target tissue.

Flexible electrode arrays based on polymeric substrates have been developed as bioelectronic surface interfaces.^[^ ^13^, ^14^, ^15^ ^]^ Such substrates can be made extremely thin and conformable and tend to ensure biocompatibility.^[^ ^16^, ^17^, ^18^, ^19^ ^]^ Yet, there have been calls for softer and more flexible interfaces for improving the integration with biological tissue and in vitro cell cultures.^[^ ^20^, ^21^, ^22^, ^23^, ^24^, ^25^, ^26^, ^27^, ^28^, ^29^ ^]^ As an example, various conductive materials have been embedded in silk fibroin (SF), such as graphene, graphene oxide, and conductive polymers.^[^ ^30^, ^31^, ^32^, ^33^ ^]^ Recent work, for instance, prepared a conductive regenerated SF scaffold, produced through electrospinning, with a graphene sheet between the microfibers, for use as an interface to PC12 cells.^[^ ^34^ ^]^ J. Ding et al. demonstrated a wearable cardiac monitor for stable epicardial ECG recordings using a 10 × 10 mm hydrogel electrode consisting of carbon nanotubes and silk.^[^ ^35^ ^]^ Expanding on these previous works, we combine carbon nanoparticles with a SF solution, producing an ink that can be inkjet‐printed to directly draw a conductive pattern at 50 µm resolution onto a silk substrate. Moreover, we tune the silk ink to be used as both, substrate and passivation layer. The manufacturing process is as follows: First, the substrate is produced by inkjet printing SF to create a homogeneous film. Next, carbon is printed onto the film to form the electrode material. A drop of the desired chemical is then printed onto the carbon. Subsequently, a SF‐insulator layer is printed on top of the carbon feed lines, leaving the carbon electrode opening exposed. Last, doped SF is printed on top of the electrode opening, covering it. Finally, we describe a new approach for an electrically controlled drug delivery system by tuning the SF ink. Modern medical treatment methods for cardiovascular diseases are creating new ways to mitigate patients’ debilitating symptoms, but current pharmacological treatments administer these medications systemically, leading to widespread drug distribution and raising the toxicity risk.^[^ ^36^, ^37^ ^]^ Administering drugs in small volumes to targeted tissue regions while minimizing leakage and diffusion would be beneficial in light of the fact that critical cell circuitry tends to involve cell‐specific characteristics and minute volumes.^[^ ^27^, ^28^ ^]^

## 2 Results and Discussion

### 2.1 Printed Silk MEA Characterization

**Figure 1:**
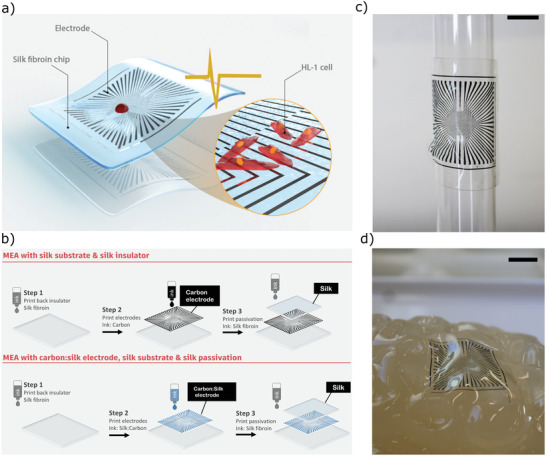
Schematic representation of a) the printed soft silk MEA and b) steps for fabricating the MEA by inkjet printing. c) Photograph of printed MEA using silk as an insulator and substrate (scale bar = 0.5 cm) wrapped onto a glass cylinder. d) Image of the silk MEA on gelatin brain model which illustrates the high degree of conformal contact that can be achieved; to allow the devices to be manually handled, the chips in both (a) and (b) were supported by 50 µm PEN foil.

The silk MEA developed in this study used a carbon electrode and carbon hydrogel electrode, as schematically presented in **Figure** **1**. First, a thin silk layer was printed as a substrate and back insulator (Examples of ≈270 nm height measurements are shown in Figure S1, Supporting Information). Then, the electrode was printed, which was either a) carbon ink or b) a carbon silk ink (see Figure S2, Supporting Information). Based on optical measurements, the electrodes had a surface area of (2.5 ± 0.4) × 10^3^ µm^2^ (*n* = 26). The reliable printing resolution is in the range of ≈30 µm as shown in previous work.^[^ ^38^ ^]^ Next, an additional layer of silk was printed to electrically isolate the connection traces from the biological milieu. Part of the carbon layer was exposed, with the terminal electrodes serving as sensing sites and creating a direct interface with the cells. This also generated bonding pads to electrically connect the electrode to the external monitoring system.

The use of inkjet printing allowed us to adjust the carbon nanoparticle content in the final film, meaning that the printed device's conductivity can be tuned, whereby a higher carbon nanoparticle content resulted in a printed device with higher conductivity. The following silk‐fibroin‐based materials are the main focus of this work:

1. Silk‐fibroin insulator: An insulator made by directly printing a SF solution. After additional processing, the silk can remain in the physiological environment for up to 1 month remaining its insulating properties.
2. Carbon–silk electrode: A high‐carbon SF solution used as a conductive hydrogel–silk electrode.
3. Doped SF film: A low‐carbon content SF solution that forms a layer that can be triggered electrically to release a drug.

**Figure 2:**
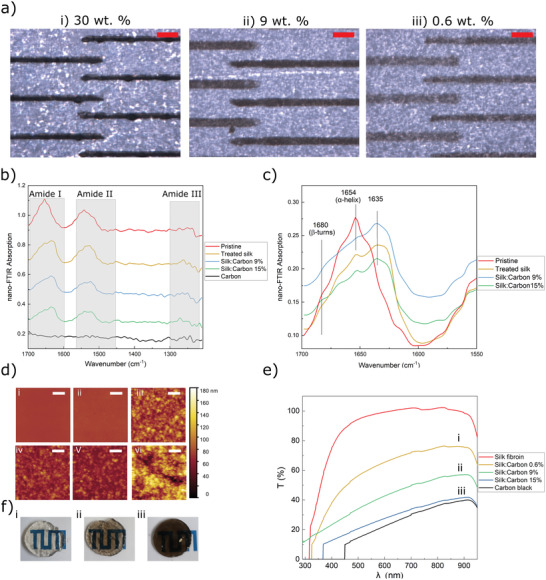
a) Microscopic images of the successive printing process of a carbon MEA using 30, 9, and 0.6 wt% carbon ink on SF back insulator, scale bars represent 200 µm. b,c) FTIR spectra in dependence of silk *β*‐sheet content of the SF films. d) i to vi: AFM topography measurements of pristine silk, treated silk, inks B, C, D, and carbon, respectively. The scale bar indicates 1 µm and the color bar applies to all the plots. e) Light transmittance of films made of different inks (thickness 10 mm). f) Optical photographs of films made of i) ink D, ii) ink C, and iii) ink A.

A test set of SF‐carbon nanoparticle composite electrodes was inkjet‐printed, whereby the proportion of the carbon nanoparticles was varied to enable a comparison through electrical and optical characterization. **Figure** **2**a shows images that were obtained after printing pure carbon ink (30 wt% of carbon nanoparticles containing no SF) and ink composed of different weight ratios of silk to carbon (Figure 2a(ii–iii)) on top of a SF back insulator. As expected, we found that the printed pattern's electrical conductivity increased with an increasing proportion of carbon nanoparticles in the ink (**Table** **1**). However, silk–carbon inks with a carbon content above 9 wt% led to poor printing performance and introduced difficulties in obtaining a homogeneous pattern. We found that the printed pattern containing 9 wt% carbon nanoparticles produced a sheet resistance of (6.3 ± 1.6) × 10^3^ Ω/□. The resistivity of our carbon films is comparable with previous reports on conductive carbon inks.^[^ ^39^ ^]^

**Table 1:** Effect of carbon black and SF content on conductivity of printed structures

Ink	Carbon [wt%]	SF [wt%]	Sheet Resistance [Ω/□]
A	30	0	(2.8 ± 0.3) × 10^2^
B	15	70	(3.8 ± 0.2) × 10^3^
C	9	70	(6.3 ± 1.6) × 10^3^
D	0.6	70	(4.4 ± 2.4) × 10^4^

Further experiments were conducted using two carbon nanoparticle proportions, termed ink C, with 9 wt% carbon, and ink D, with 0.6 wt% carbon. The next section presents the experiments using ink C as a recording hydrogel electrode and ink D as the drug delivery composite. The printed MEAs' other characteristics, including the results of the mechanical and electrochemical evaluations, are given in the Supporting Information (Figure S3, Supporting Information).

### 2.2 Near‐Field Fourier Transform Infrared Spectra

SF shows superior properties over other biomaterials due to its biocompatibility and water solubility. Moreover, its biodegradability can be programmed to last any time from hours to years through the modification of the processing procedures or postprocessing treatments. SF consists of *α* helices, *β*‐sheet crystals, and random coils, created by repetitive amino acid sequences produced through hydrogen bonds, hydrophobic interactions, and van der Waals forces. Processes such as water vapor annealing, ethanol annealing, and methanol annealing primarily alter the *β*‐sheet crystal content or the organization degree of the non‐crystalline domains. For example, SF biodegradability can be accelerated by decreasing the *β*‐sheet crystal content. Inversely, increasing the *β*‐sheet crystal content can enhance its mechanical properties.^[^ ^40^ ^]^

To investigate the changes in the crystallinity of the SF material following different treatments, near‐field Fourier transform infrared spectra (nano‐FTIR) and AFM measurements were performed. Figure 2b,c shows nano‐FTIR absorption spectra, measured on pristine silk (printed SF without any post‐treatment), treated silk (SF after water vapor annealing), printed ink A, ink C, and ink D. Peaks can be seen at 1635 and 1680 cm^−1^, which are assigned to the *β*‐sheet and *β*‐turn. These demonstrate the increasing intensity with increasing treatment time, implying increases in *β*‐sheet and *β*‐turn. There is a broad peak visible from 1635 to 1665 cm^−1^, which is assigned to either random coil or helical conformation, or both (for amide I).^[^ ^41^ ^]^ AFM topography measurements of 4 × 4 µm^2^ films of pristine silk, treated silk, inks B, C, D, and carbon are presented in Figure 2d(i–vi), respectively. Pristine and treated silk exhibit minimal roughness, while the presence of carbon leads to a remarkable increase in surface roughness. The higher the ratio of carbon, the larger the roughness of the surface. The height variation ranges in these AFM scans start from 4 nm for pristine silk and rise up to 180 nm for carbon.

Figure 2f shows the transmission spectra of SF film made from different inks at a fixed layer thickness; the transmittance spectra of carbon layer and SF substrates are also shown for comparison. The annealed SF film exhibits maximum transmission. As the ratio of the black carbon increased, the nanocomposite film became opaque at wavelengths above 450 nm. Compared with carbon electrodes, silk–carbon nanocomposite electrodes exhibit a higher transmittance especially for 0.6 wt% of carbon (ink D). The photographs of silk–carbon nanocomposite from different inks (B, C, and D) are shown in Figure 2e(i–iii), which shows that ink C has a high transparency for use as conductive semitransparent electrode.

### 2.3 Silk as an Insulator

**Figure 3:**
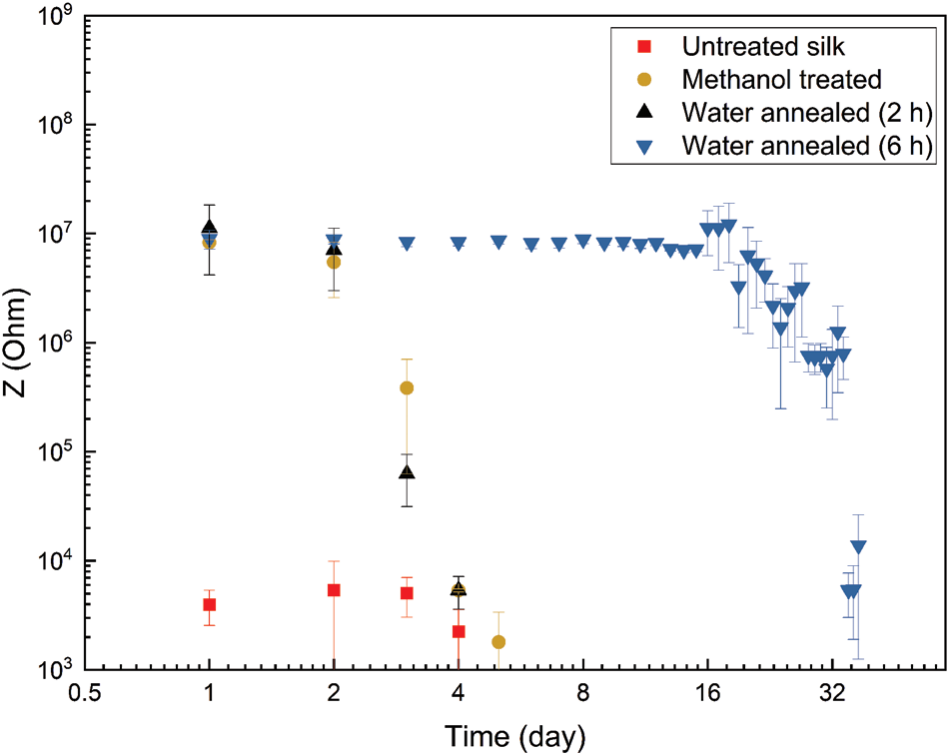
Impedance measurement at 1 KHz of printed carbon microelectrodes fully covered by silk insulator exposed to cell medium over 37 days (the mean and standard deviation were calculated using ten printed microelectrodes).

We assessed the printed SF in terms of the durability of its insulating properties following water annealing. Hereby, the carbon electrodes were fully passivated with a printed layer of SF, and then measured by electrochemical impedance spectroscopy (EIS) for 37 days in a physiological solution at 37 °C. **Figure** **3** illustrates the impedance changes at 1000 Hz versus time as a measure of the electrode impedance stability, whereby the material was treated with methanol annealing for 6 h, water annealing for 6 h, or no post‐treatment (control). The figure clearly shows that the electrode impedance stability increased significantly after water annealing for 6 h. Based on this result, water annealing for 6 h was considered suitable to ensure stability during extracellular recordings.

### 2.4 Electro‐Responsive Silk

**Figure 4:**
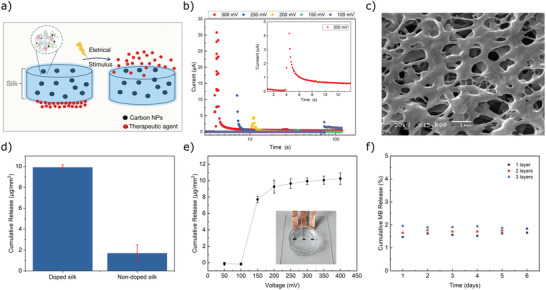
Trigger and release behaviors of electro‐responsive printed silk carbon nanocomposites. a) Schematic representation of drug delivery concept. b) Chronoamperometry measurements using printed silk carbon nanocomposite. c) SEM image after voltage pulse application. d) Total methylene blue release from silk films with and without carbon nanoparticle content in response to an aggressive voltage stimulation (0.5 V for 5s). e) Cumulative release profile of the methylene blue in response to voltage stimulation, error bars represent standard deviation from the mean (*n* = 6). The profile reaches a plateau at 250 mV. f) Average release profile (*n* = 4) over 6 days without applying any voltage measured for different numbers of printed layers (1–3).

Recent studies have reported several systems able to store drug in a chemically conjugated polymer structure or a porous polymer molecular matrix, with drug release being triggered by a thermal stimulus delivered wirelessly.^[^ ^42^, ^43^, ^44^ ^]^ In contrast, we precisely print the drug at a picoliter volume, sandwiching it between a carbon electrode and an electro‐responsive silk–carbon layer, rather than directly incorporating it into the silk polymer. Consequently, the drug does not need to be incorporated via a drug–hydrogel interaction, but rather the electro‐responsive silk–carbon layer acts as a seal that is released by a voltage‐based trigger. Our concept of an electro‐responsive drug delivery system based on SF is schematically presented in **Figure** **4**a, whereby the desired drug is sandwiched between a carbon electrode and a silk–carbon nanocomposite. Briefly, a layer of a compound (in this paper, fluorescent nanoparticle or methylene blue) is printed onto the opening of a previously printed carbon electrode; this opening is subsequently sealed with a drop of an electro‐responsive silk–carbon nanocomposite. Drug release can then be initiated by applying a voltage to the electro‐responsive layer. Chronoamperometry technique was used to elucidate the relationship between the applied voltage and the time needed for layer opening, as shown in Figure 4b. The applied voltage leads to an opening in the layer made of ink D, as demonstrated by a sudden increase in the current. This is followed by a stable region in which the current is higher than the current at the beginning of the applied voltage indicating an increase in the electrode's active area due to removal of the silk carbon nanocomposite. SEM was used to determine the morphology of the film, which has been exposed to 300 mV for 5 s. As shown in Figure 4c, a highly porous layer with pore sizes in the range of 1 µm was observed. A SEM of printed SF nanocomposite before applying a voltage stimulus is shown in Figure S4, Supporting Information. This porous structure, which is formed upon electrical stimulus of the electrode will facilitate the diffusion of chemicals or a therapeutic agent when the film is used for drug delivery on a chip. We suggest that the application of an electric field to the silk–carbon nanocomposite induces local breakdowns in the film that triggers morphological changes in the conducting polymer network. Instabilities in soft polymeric films due to high electric field strength have been described before.^[^ ^45^, ^46^ ^]^ Here, the conductive carbon filler material could effectively reduce the length of dielectric bridges leading to an effective increase of local electric field strength and local breakdown.

We evaluated the drug delivery performance of the silk carbon nanocomposite film (printed using ink D) via an experiment using methylene blue as model compound with an absorbance peak at 662 nm. A calibration curve was constructed with UV–vis absorption. First, a layer of methylene blue was printed on top of carbon macroelectrode. Next, a silk carbon nanocomposite film was inkjet‐printed to cover the methylene blue layer. The nanocomposite was immersed in phosphate‐buffered saline (PBS) and subjected to voltage pulse of 0.5 V for 5 s. UV absorbance spectroscopy was then used to analyze the solution, showing how much methylene blue had been released. The drug release from conventional silk films that were not doped with carbon was also assessed for comparison (Figure 4d). The nanocomposite released ≈6× the amount of the chemical compared to the SF films without carbon doping.

To identify the maximum amount delivered by the silk carbon nanocomposite film, we used different voltages to assess the cumulative release. No methylene blue could be detected under 0.15 V, and a plateau was reached after 0.3 V, indicating that the film could not release any more of the methylene blue after that level (Figure 4e). We also monitored the chip over several days without applying any voltage pulse and found no significant methylene blue release, indicating the absence of diffusion of the chemical from the bare film (Figure 4f). The amount of methylene blue delivered by the nanocomposite could be altered by modulating the voltage stimulation magnitude, highlighting that this drug delivery system offers dosage control and flexibility. Our silk carbon nanocomposite film demonstrates a monotonic increase in the cumulative release with the pulse voltage, making it an excellent candidate as a long‐term (7 days) yet temporally precise drug delivery system.

**Figure 5:**
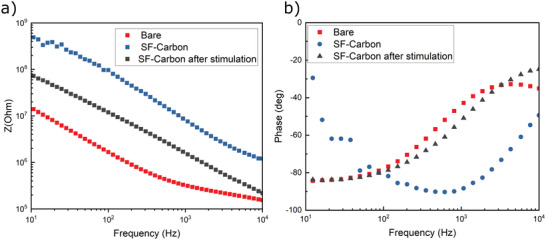
Electrochemical properties of the silk carbon nanocomposite film. Electrochemical impedance spectra recorded in PBS using macroscopic films of bare carbon electrodes, electrodes modified with a silk carbon nanocomposite, and electrodes after voltage trigger.

Electrochemical Impedance Spectroscopy (EIS) was used on control structures to examine the carbon–silk nanocomposite film's electrical properties, specifically to show how coatings affect the electrode's impedance across a broad frequency range. **Figure** **5** presents the impedance, performing a comparison between a conventional carbon electrode, a carbon electrode coated with the carbon–silk nanocomposite film, and the same electrode following the application of a voltage trigger. The carbon–silk film coated electrode shows increased impedance for all measured frequencies over the bare electrode, with the coating acting as a barrier. Furthermore, the impedance drops if a voltage (0.3 V) is applied, suggesting a perforation of the barrier, that is, the carbon–silk film opens to release the underlying chemical.

### 2.5 Observation of Triggered Fluorescein Release on Microelectrodes

**Figure 6:**
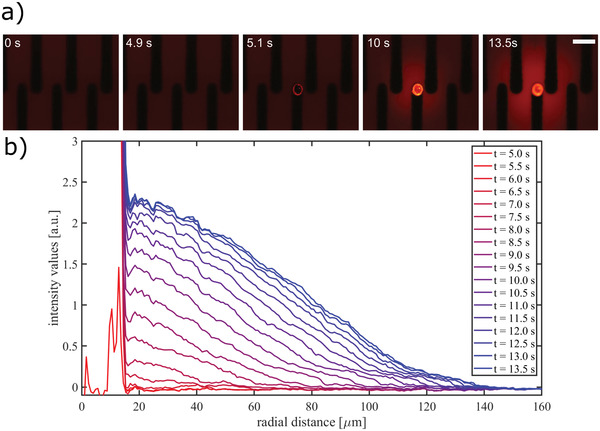
Fluorescein nanoparticle release during electrical stimulation. a) Fluorescence imaging snapshots of the release event with the corresponding timestamp. The trigger is applied at 0 s. (scale bar 100 µm). b) Fluorescence intensity values as a function of radial distance from the center of the droplet. The background image is subtracted from the frames, and the values are integrated over the angles.

In order to demonstrate film functionality, a proof‐of‐concept experiment was designed to explore whether the voltage pulse triggered release of fluorescein nanoparticles. First, we directly printed a single drop of fluorescein nanoparticles (diameter of 100 nm) onto a carbon microelectrode. This was then covered by a drop of the carbon–silk nanocomposite film. Afterward, we used fluorescence microscopy to image the release of the fluorescein following electrical stimulation, which enabled the quantification of the amount of fluorescein released. **Figure** **6** depicts the release event of the fluorescein nanoparticles, followed by their diffusion. Although the electric potential is applied continuously since the beginning of the recording, first signs of fluorescence are evident at *t* = 5 s. As seen in Figure 6a,b, the release is predominantly initiated along the edges of the disk. The release and the diffusion of the fluorescein nanoparticles continue throughout the measurement.

### 2.6 HL‐1 Cellular Recording Using Silk MEA

**Figure 7:**
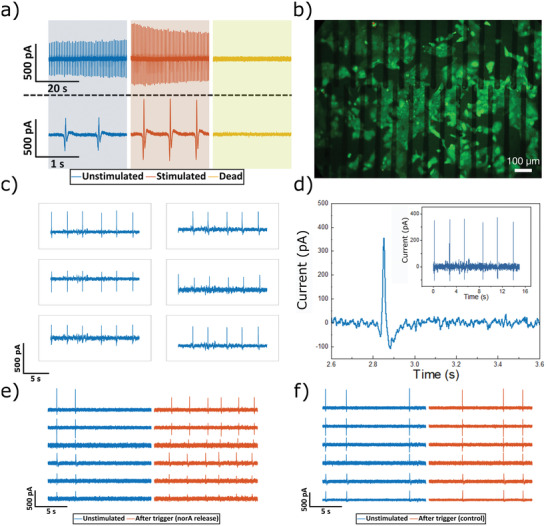
Printed MEA using silk hydrogel substrates and passivation for extracellular recording. a) Action potential recordings where initially cells are firing at a frequency of 0.3 Hz (blue), whereas addition of noradrenaline into the culture medium increased the beating frequency (red) and SDS starts to perforate and dissolve the cellular layer leading to stopping the beating of the cells (yellow). b) Fluorescence microscopy image of live/dead staining of HL‐1 cells growing on a silk MEA (scale bar 100 µm). c,d) Action potential recordings from HL‐1 cells using printed hydrogel electrode. e) Action potential recordings before and after the voltage trigger had locally released the printed noradrenaline. f) The voltage trigger's effect on HL‐1 cells cultured without noradrenaline under the electro‐responsive silk–carbon layer.

Extracellular recordings were made from cardiomyocyte‐like HL‐1 cells (**Figure** **7**) to assess the silk MEA in terms of functionality. First, the cells were cultured for a few days on the MEA to produce a confluent cell layer. The initiation of spontaneous contractions confirmed that the printed MEA were compatible with a layer of active cells. The cells’ action potential generation was monitored locally via amperometric recording on a homemade amplification system that had the capacity to record on up to 64 channels at a 10 kHz sampling rate per channel.^[^ ^47^ ^]^ The recording in Figure S5, Supporting Information, presents a typical 20 s segment of spontaneous activity of the HL‐1, cells taken using 64 electrodes. The channel with the maximum peak‐to‐peak (P2P) amplitude showed an amplitude of 980 ± 50 pA (mean of all P2P values ± standard deviation (STD) of all P2P values within one trace). The maximum signal‐to‐noise ratio (SNR) was 34, with the signal value of 810 ± 40 pA and the noise level of 24 pA. The noise was calculated as the STD of spike‐free traces. After an automatic spike detection, from 30 ms before until 70 ms after each spike was ignored in the STD calculation. With this definition, 15% of the channels showed SNR values above 15 which is in line with previous findings on HL‐1 recordings made via printed MEAs adhering to flexible substrates.^[^ ^48^ ^]^ To investigate the recorded signals’ specificity, we used noradrenaline (NA), a catecholamine, which induces a sympathetic response, as a chemical trigger for the cells. In line with our expectations, adding 2 µL of 10 mm NA led to a rise in the spontaneous action potential firing rate from 1.3 to 1.8 Hz. Once the measurements had been completed, sodium dodecyl sulfate (SDS) was added to confirm the signal and terminate all cells (Figure 7a).

Figure 7b shows cell viability using fluorescent live–dead staining. In order to demonstrate the versatility of our approach for using silk–carbon nanocomposite as a conductive hydrogel electrode on soft materials, we explored MEA printed using ink C on silk substrates (see Figure 7c). The functionality of the MEAs was evaluated by performing cellular recording of action potentials. Figure 7d shows an exemplary trace from the same MEA. As a proof‐of‐principle, we applied the drug delivery approach in connection with on‐chip cell recording. To this end, devices were inkjet‐printed with noradrenaline‐containing ink between a carbon recording electrode and our electro‐responsive silk–carbon layer in a sandwich‐type design. Hl‐1 cells were then cultured on these devices without any noradrenaline in the medium resulting in an irregular beating pattern. Figure 7e shows action potentials recorded from the Hl‐1 cells before and after a voltage were applied to induce noradrenaline release. To provide a control, the same devices were printed without noradrenaline and the same voltage pulse was applied. The comparison revealed that devices with noradrenaline displayed increased beating frequency as illustrated in Figure 7f.

For future work, rather than using glass to support the ultrathin MEA for in vitro applications, we envision that a biodegradable material such as gelatin or silk could serve as a temporary stiffener for in vivo applications.

## 3 Conclusion

This work describes a novel silk‐based MEA that is both soft and ultraflexible. The MEA is a sandwich construction of a silk–carbon nanocomposite in between a substrate and an insulator, both also made of silk. We have demonstrated how layers with various properties, including conductive, insulating or electro‐responsive characteristics, can be achieved by varying the composition of the silk ink. When used as an insulator in a physiological environment the silk barrier showed good stability over 30 days. Using models with methylene blue and fluorescein nanoparticles, we examined the drug release behavior of this nanocomposite in the form of macro and microelectrodes, demonstrating that by tuning its properties it can serve as a drug‐delivery platform that can be controlled electrically. Such a precise technique for on‐demand drug delivery would pave the way for effective therapies with reduced toxic effects as the drug delivery could be adjusted to meet the temporal and spatial requirements of specific conditions. Moreover, in vitro assays may also benefit from such a controlled delivery, for example, when performing high‐volume drug screening or as part of cell biology investigations. Because the properties of the silk nanocomposite can be adjusted, facilitating both stability and dosing control, the platform described here may contribute to the development of new drug delivery techniques based on customized drug‐release profiles. Last, the silk MEAs were shown to be applicable for the in vitro extracellular recording of the action potential of HL‐1 cells. Future work should consider using a biodegradable silk layer as a temporary stiffener for the implementation of devices for in vivo applications.

## 4 Experimental Section

### Carbon Ink Preparation

The process of preparing the carbon ink was done as in prior work.^[^ ^29^ ^]^ To summarize, 1 g of carbon black (Orion Engineered Carbons, Luxembourg) was added to 5 g of ethylene/glycol (50/50 wt%) mixture. The resulting mixture was milled for 1 h at 1100 rpm using 100 µm yttrium zirconium beads and a Pulverisette 6‐ball mill (Fritsch, Germany). Then, 10 g of the ethylene glycol/water mixture was further added to dilute the resulting mass, and the mixture was thoroughly stirred to obtain the desired viscosity. After this, the mixture was supplemented with 0.2 wt% polyacrylic acid (Sigma‐Aldrich, 35 wt% solution) to ensure that the ink would adhere to the substrate. Last, the final ink was produced by passing the mixture through a 0.45 µm filter.

### Inkjet Printing

A Dimatix Materials Printer (Dimatix DMP‐2850, Fujifilm Dimatix Inc., USA) was used to produce the MEAs. The inks were first prepared for printing by sonicating them for 5 min, and then they were passed through a 0.2 µm PVDF syringe filter. The following parameters were employed in the printing process: 2.4 pL Samba cartridges (Fujifilm Dimatix Inc., USA), a 1 kHz jetting frequency, a 22 µm drop spacing, a substrate holder and printhead temperature of 21 °C, a 16 µs jetting period, and a 40 V jetting voltage. For the planar substrates, the printhead‐substrate distance was 250 µm. To ensure the easy handling of the ultra‐flexible MEA, SF was jetted onto glass substrates or 50 µm PEN foil. The noradrenaline ink (NA ink) consisted of 60 wt % NA solution (10 mm NA dissolved in 30 mm ascorbic acid) and 40 wt % dipropylene glycol. Fluorescent nanoparticle ink was prepared by mixing equal ratio of 0.1 µm FluoSpheres carboxylatemicrospheres (Invitrogen, Thermo Fisher, Germany) and dipropylene glycol.

### Preparation of SF Proteins

Aqueous SFn solution was prepared using the established purification protocol.^[^ ^49^, ^50^ ^]^ Briefly, silk cocoons derived from Bombyx mori silkworms (Satoyama Craft, Fukushima, Japan) were degummed for 30 min in a 20 mm solution of boiled sodium carbonate (Carl Roth, Germany) for removing silk sericin, rinsed with distilled water, and dried in a desiccator overnight. The degummed and extracted SF fabrics were dissolved in a 9.3 m lithium bromide solution (Sigma‐Aldrich, Germany) for 3.5 h. This was followed by dialysis against the distilled water using dialysis cassettes with a 3500 molecular weight cutoff (Slide‐a‐Lyzer G3, Thermo Fisher Scientific, USA) to convert the solvent to an aqueous 60 mg mL^−1^ solution of SF.

### Electrically Controlled Drug Release

For all release experiments of electro‐responsive printed silk carbon nanocomposites, modified electrodes were immersed in PBS and submitted to release stimulation. The PBS solutions containing the released methylene blue were analyzed with UV–vis spectroscopy (Analytik Jena Specord 200 [Jenoptik, Germany]) at a wavelength of 662 nm to quantify the amount of methylene blue released. To determine the cumulative release of the methylene blue in response to voltage stimulation, the release solution was removed for analysis at given voltage pulses was collected and measured using UV–vis spectroscopy.

### Nano‐FTIR Spectroscopy

All the measurements were carried out in ambient atmosphere using a commercial s‐SNOM in reflection mode (neaSNOM, attocube Systems AG). A PtIr‐coated AFM tip (Arrow‐NCPt, NanoAndMore GmbH) was illuminated using a coherent, broadband pulsed beam from a difference frequency generation laser source (TOPTICA Photonics AG). The output beam was set to the 1200–2200 cm^−1^ spectral range, with an integral power of <1 mW. The scanning was performed in tapping mode with the amplitude of about 100 nm at the frequency of ≈250 kHz, thus modulating the scattered light at this frequency and its higher harmonics. The backscattered light was analyzed with a Michelson interferometer in asymmetric configuration, where the sample and the tip were positioned in one arm of the interferometer. Demodulation of the scattered light at the second harmonic of the tip was used to separate the contribution of the near‐field signal from the background. Near‐field spectra were normalized by a reference spectrum recorded on a clean portion of the Si substrate. Scan data was leveled with Gwyddion 2.59. Nano‐FTIR data was acquired with 600 µm of interferometer travel distance, 2048 pixels per scan, 10 ms of integration time per pixel, and four scans averaged per point.

### Electrical and Electrochemical Characterization

All chips were incubated with deionized water for 5 min for cleaning before the electrochemical measurements were performed. A reservoir was produced by adhering the MEAs to glass rings 10 mm high and with a 7 mm diameter using PDMS. A Biological potentiostat (VSP‐300 potentiostat from BioLogic Science Instruments) was utilized to conduct the electrochemical experiments. The medium for all experiments was PBS as a supporting electrolyte. An Ag/AgCl electrode (Super Dri‐Ref SDR 2; World Precision Instruments, USA) was used to reference the recorded signals.

The sheet resistances were measured using a four‐point probe head from Jandel connected to a Keysight B2900A source measuring unit.

### Cellular Recording

The MEAs were incubated in 70% ethanol for 10 min for sterilization, after which they were rinsed using sterile distilled water. Next, a coating comprising 2.5 µg cm^−2^ fibronectin from bovine plasma (Sigma‐Aldrich, Germany) was applied over 1 h in calcium and magnesium‐free PBS (Life Technologies GmbH, Darmstadt, Germany) at 37 °C. Prior to cell seeding, the chips were subjected to a single rinsing with supplemented Claycomb medium (Sigma‐Aldrich, Germany). The cardiomyocyte‐like HL‐1 cells were kept in a Claycomb medium that was supplemented with 10 vol% fetal bovine serum (Life Technologies GmbH, Germany), 100 µg mL^−1^ penicillin–streptomycin (Life Technologies GmbH, Germany), 0.1 mm (±)‐norepinephrine (+)‐bitartrate salt (Noradrenaline, Sigma‐Aldrich, Germany), and 2 mm l‐glutamine (Life Technologies GmbH, Germany). A humidified incubator was used to keep the cells at 37 °C and 5% CO_2_. Every day, the medium was replaced with fresh medium. When confluency was achieved, the contracting cell layer was first washed and then detached by incubation with 0.05% trypsin‐EDTA (Life Technologies GmbH, Germany) at 37 °C. Supplemented Claycomb medium was then added to halt the trypsin digestion, and the cells were centrifuged at 200 rcf for 5 min to achieve sedimentation. Resuspension of the cells was done in pre‐warmed, supplemented Claycomb medium. 100 µL of the suspension was then applied to the center of each chip. The chips were then placed in a humidified incubator for 30 min at 37 °C and 5% CO_2_ to promote adhesion. Last, each chip was placed in 500 µL of medium, which was exchanged every day, until confluency was achieved. When a confluent cell layer was observed beating (within ≈2 days), a 64‐channel transimpedance amplifier system was used to measure the action potentials as described in prior work.^[^ ^47^ ^]^ For the drug release experiments, on the day of the cell recording, the cells were fed with noradrenaline free medium and measurements were taken 6 h after changing the medium.

For the statistical assessment of the results presented (HL‐1 cellular recording using silk MEA), MATLAB's findpeaks* function was employed to detect the individual spikes having a minimum peak prominence of six times the standard deviation of the trace. To calculate the background noise, the regions with spikes were ignored and the standard deviation was calculated on the remaining data.

## Conflict of Interest

The authors declare no conflict of interest.

## Supporting information

Supporting Information

## Data Availability

The data that support the findings of this study are available on request from the corresponding author. The data are not publicly available due to privacy or ethical restrictions.
