# Supplementary material for: Printed Silk Microelectrode Arrays for Electrophysiological Recording and Controlled Drug Delivery
Source: Adv Healthc Mater. 2023 Mar 3;12(17):2202869. doi: 10.1002/adhm.202202869 (PMC11468847; doi:10.1002/adhm.202202869)
Supplement: Supplementary file 1 — Supporting Information [file ADHM-12-2202869-s001.pdf]

ADVANCED HEALTHCARE MATERIALS

Supporting Information

for *Adv. Healthcare Mater.*, DOI 10.1002/adhm.202202869

# Printed Silk Microelectrode Arrays for Electrophysiological Recording and Controlled Drug Delivery

*Nouran Adly, Tetsuhiko F. Teshima, Hossein Hassani, George Al Boustani, Lennart J.K. Weiß, Gordon Cheng, Joe Alexander and Bernhard Wolfrum**

Supporting Information

**Printed silk microelectrode arrays for electrophysiological recoding and controlled drug delivery**

*Nouran Adly, Tetsuhiko F. Teshima, Hossein Hassani, George Al Boustani, Lennart K. Weiß, Gordon Cheng, Joe Alexander, Bernhard Wolfrum**

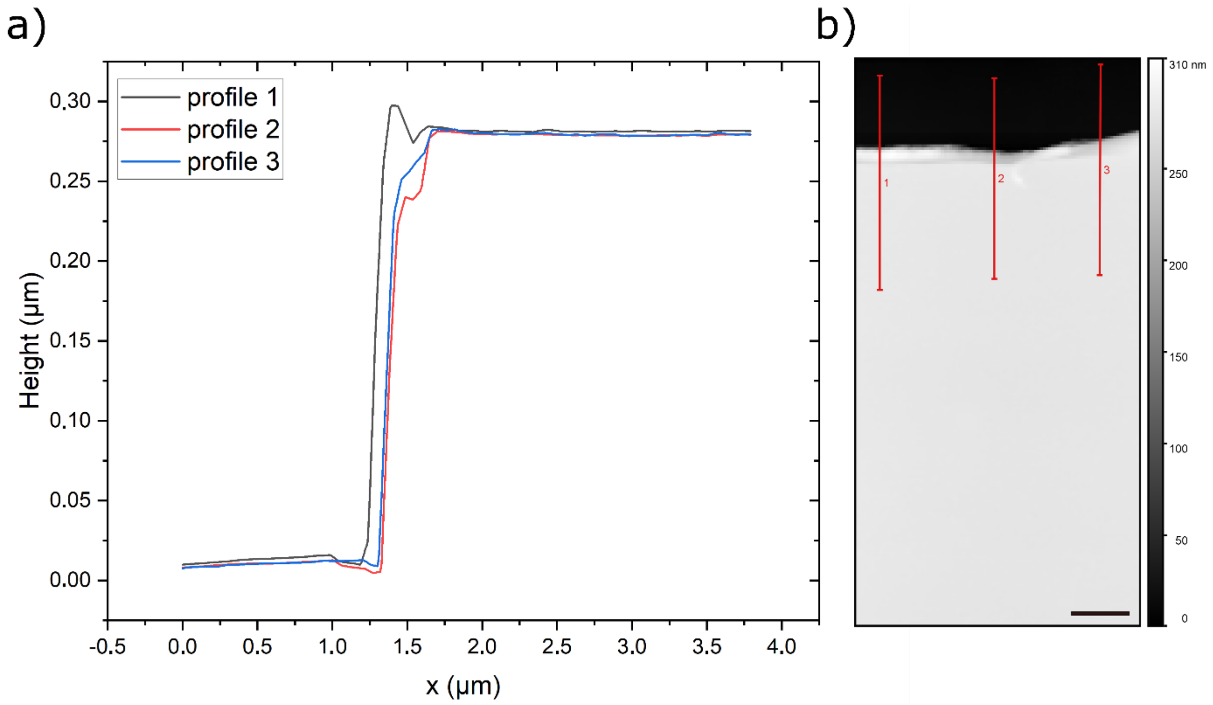


**Figure S1: a)** height profiles of untreated silk layer vs. the substrate, taken along the lines indicated in **(b)**, the AFM height map. (scale bar = 1 µm)

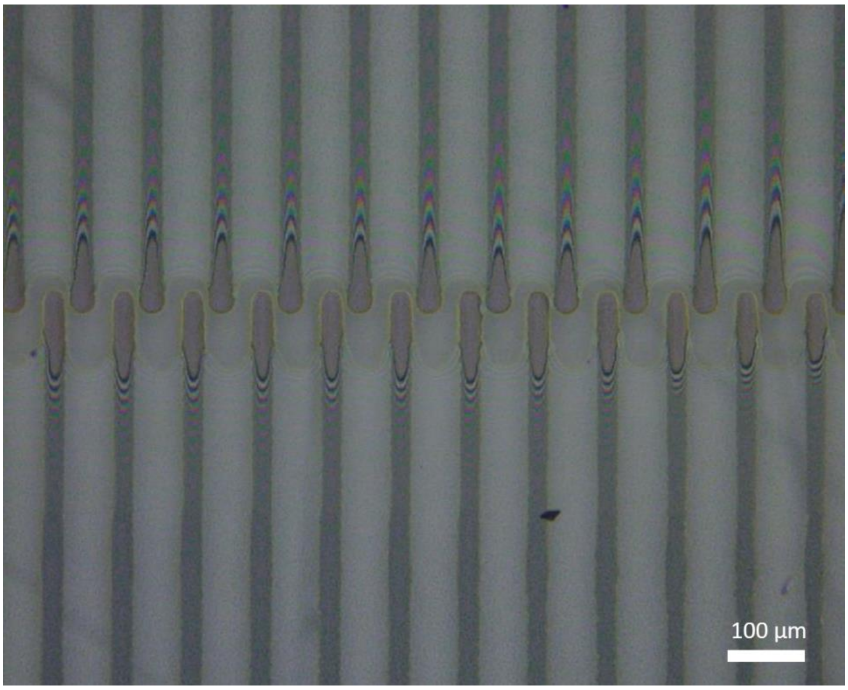


**Figure S2**: Microscopic image of the printed MEA on a silk substrate (scale bar: 100 µm)

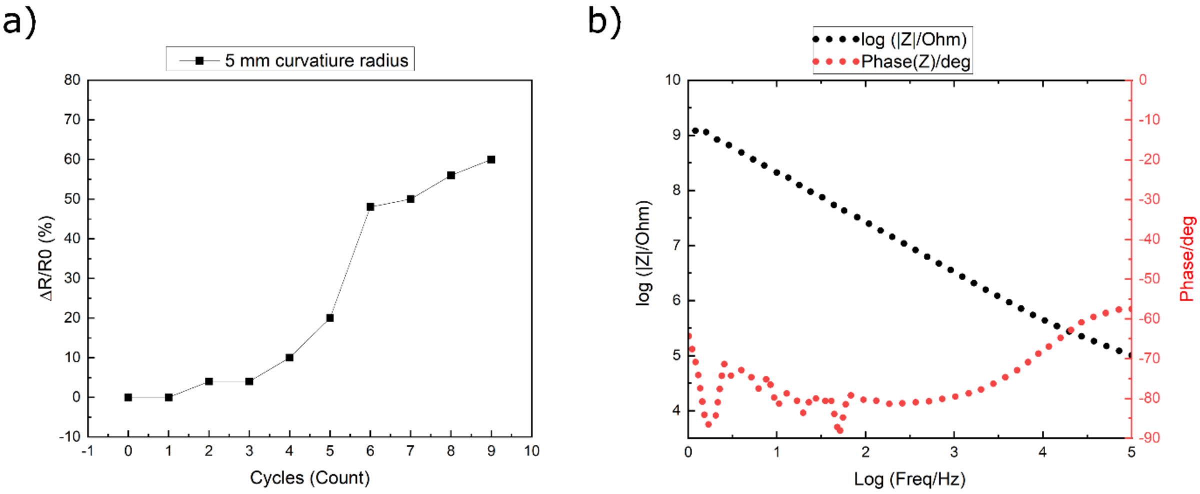


**Figure S3: a)** The bending stability of the silk-carbon electrodes; relative resistance as a function of bending cycles to 5 mm radius of curvature. b) Electrochemical impedance spectroscopy of a carbon microelectrode measured in PBS (electrode area ~$2.5\times10^3$ µm$^2$).

**Characterization of printed microelectrode arrays:**

To assess whether the printed microelectrode devices remained stable upon bending, we measured the resistance of printed lines after repetitive bending experiment cycles. Hereby, the substrates were bent to a radius of 5 mm and then relaxed; this cycle was repeated several times, after which electrical characterization was performed. **Figure S3a** presents the relative resistance change, $\Delta R/R_o$, where $\Delta R = R - R_o$, with $R$ and $R_o$ being the resistance of the structure when bent and relaxed, respectively. As presented in **Figure S3a**, after a bending radius of 5 mm had been achieved over 4 cycles, a 10% increase in resistance was observed.

**Figure S3b** presents the typical Bode spectra measured in PBS for a printed silk carbon microelectrode.

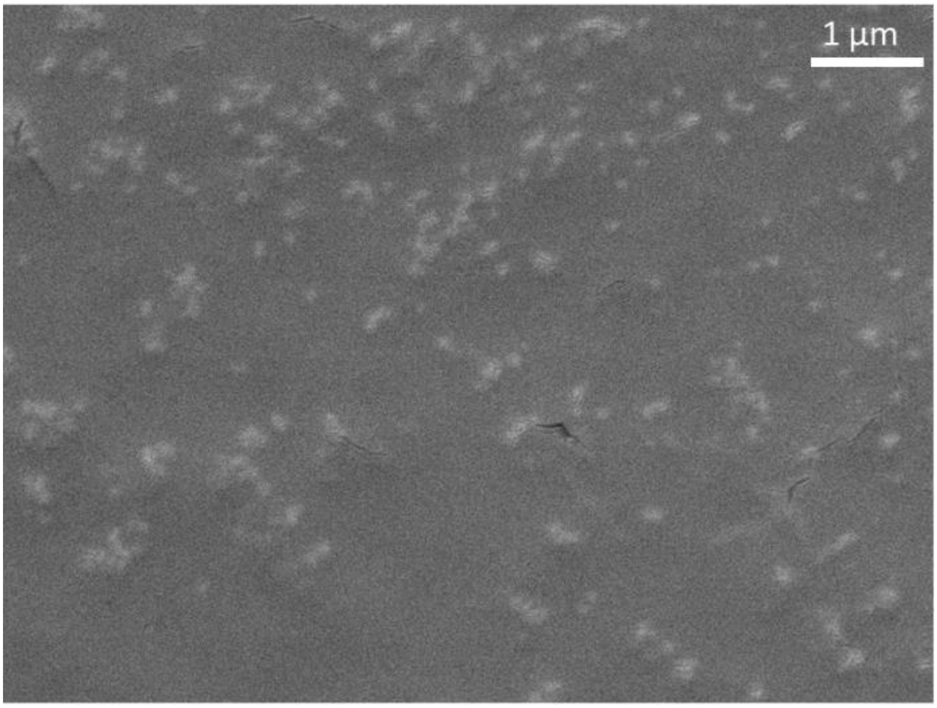


**Figure S4**: SEM image of printed SF-Carbon film without applying voltage

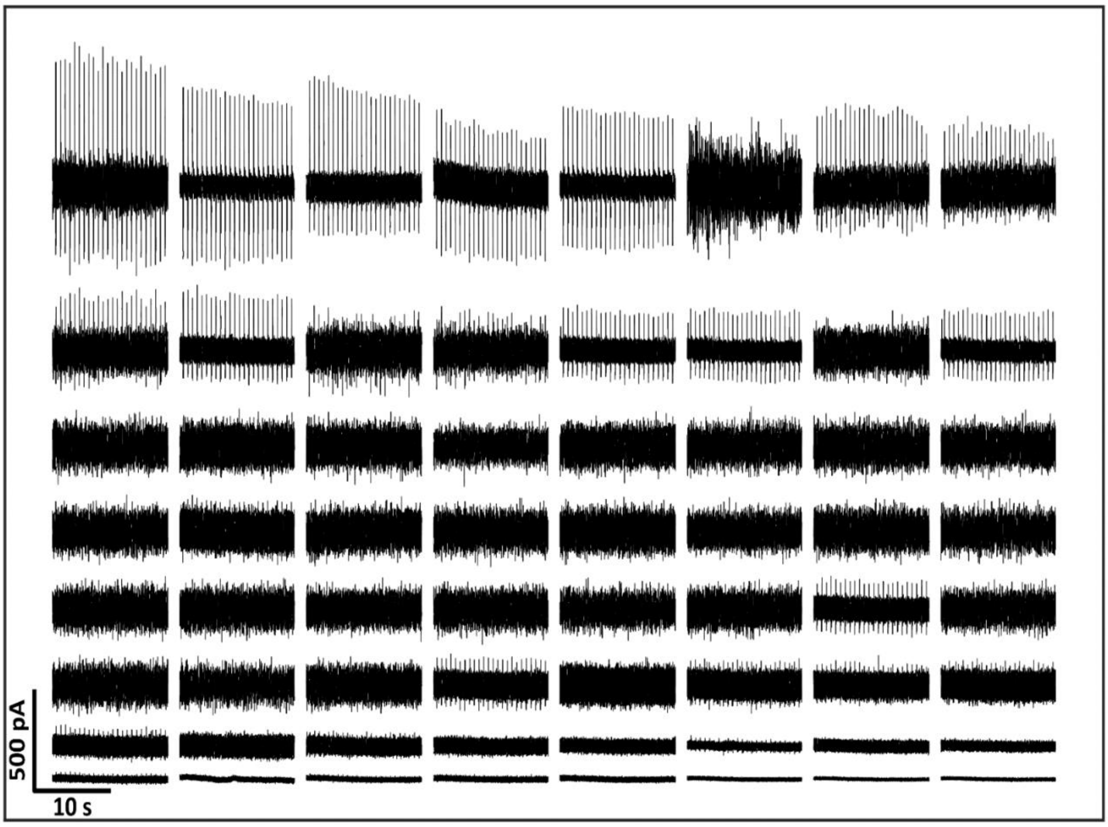


**Figure S5:** A representative recording from 62 recording channels of a single chip; recordings are organized according to descending signal amplitude.
